# Can medio-lateral baseplate position and load sharing induce asymptomatic local bone resorption of the proximal tibia? A finite element study

**DOI:** 10.1186/1749-799X-4-26

**Published:** 2009-07-17

**Authors:** Bernardo Innocenti, Evelyn Truyens, Luc Labey, Pius Wong, Jan Victor, Johan Bellemans

**Affiliations:** 1 European Centre for Knee Research, Smith & Nephew, Leuven, Belgium; 2 Department of Orthopaedic Surgery, Catholic University Leuven, University Hospital Pellenberg, Pellenberg, Belgium; 3 AZ St-Lucas, Bruges, Belgium

## Abstract

**Background:**

Asymptomatic local bone resorption of the tibia under the baseplate can occasionally be observed after total knee arthroplasty (TKA). Its occurrence is not well documented, and so far no explanation is available. We report the incidence of this finding in our practice, and investigate whether it can be attributed to specific mechanical factors.

**Methods:**

The postoperative radiographs of 500 consecutive TKA patients were analyzed to determine the occurrence of local medial bone resorption under the baseplate. Based on these cases, a 3D FE model was developed. Cemented and cementless technique, seven positions of the baseplate and eleven load sharing conditions were considered. The average VonMises stress was evaluated in the bone-baseplate interface, and the medial and lateral periprosthetic region.

**Results:**

Sixteen cases with local bone resorption were identified. In each, bone loss became apparent at 3 months post-op and did not increase after one year. None of these cases were symptomatic and infection screening was negative for all. The FE analysis demonstrated an influence of baseplate positioning, and also of load sharing, on stresses. The average stress in the medial periprosthetic region showed a non linear decrease when the prosthetic baseplate was shifted laterally. Shifting the component medially increased the stress on the medial periprosthetic region, but did not significantly unload the lateral side. The presence of a cement layer decreases the stresses.

**Conclusion:**

Local bone resorption of the proximal tibia can occur after TKA and might be attributed to a stress shielding effect. This FE study shows that the medial periprosthetic region of the tibia is more sensitive than the lateral region to mediolateral positioning of the baseplate. Medial cortical support of the tibial baseplate is important for normal stress transfer to the underlying bone. The absence of medial cortical support of the tibial baseplate may lead to local bone resorption at the proximal tibia, as a result of the stress shielding effect. The presence of a complete layer of cement can reduce stress shielding, though. Despite the fact that the local bone resorption is asymptomatic and non-progressive, surgeons should be aware of this phenomenon in their interpretation of follow-up radiographs.

## Background

One of the major failure mechanisms in total knee arthroplasty (TKA) is aseptic loosening of the tibial component. In the past this has been attributed to the quality of the fixation as well as to the strength of the supporting bone, which is subject to a more or less pronounced stress shielding effect of the proximal tibial metaphyseal bone by the tibial baseplate [1-7].

**Figure 1:**
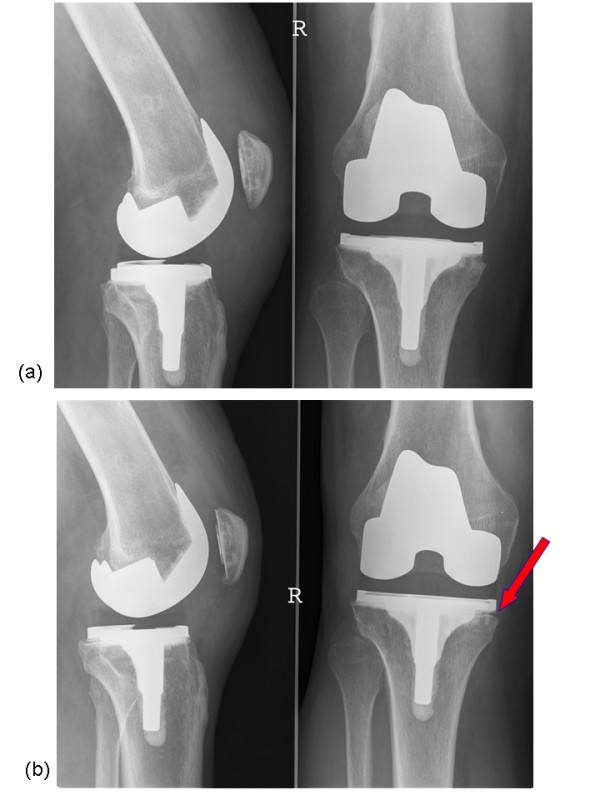
**Patient with asymptomatic focal osteolysis of the proximal tibia**. (a) Radiographs demonstrating well fixed components and normal bone-implant interface 3 months after surgery. (b) asymptomatic focal osteolysis of the proximal tibia under the prosthetic component at 1 year followup.

Asymptomatic local bone resorption of the proximal tibia under the prosthetic component can occasionally be observed after TKA (Figure 1). Its occurrence is not well documented in the literature and we are not aware of any publication that provides a clear explanation for this phenomenon. Its observation during postoperative follow-up is usually concerning to the surgeon since its significance is not well understood. In literature two main reasons for bone resorption can be found:

- Wear particles, which can induce focal osteolysis [8-13];

- Non-physiological loading conditions, mainly due to malalignment and malpositioning of the prosthesis [1-3,14-18]. In our cases the first hypothesis can be rejected because the observed bone resorption became apparent as soon as three months post operatively [11,19-21].

In this study we report the incidence of this 'short term' local bone resorption, and we investigate whether it can be attributed to a stress shielding effect, which might lead to more generalized bone resorption in the long term and potential aseptic loosening.

The overall stress distribution in a prosthetized tibia has been examined using FEA in the past already. Several parameters that can influence the stress distribution like design, material properties and fixation technique of TKA implants have been investigated. The loading conditions at the tibio-femoral interface and the implant position, may also affect the stress distribution (particularly locally), but these parameters have not been thoroughly examined [1,5-7]. For this reason, this study analyzes the possible local effects of mediolateral load distribution and implant positioning on the mechanical stress in several regions of a prosthetized tibia.

## Methods

The postoperative radiographs, made at 3 months and 1 year follow-up, of 500 consecutive TKA patients performed at our institution were analyzed to determine the incidence of local bone resorption under the tibial baseplate. All the analyzed radiographs were made under fluoroscopic guidance to ascertain true horizontal alignment of the baseplate.

All the surgical procedures were performed by the same surgeon using and the same surgical approach. All cases had undergone a standard posterior stabilized TKA using cemented fixation of either the Genesis II or Profix System (Smith & Nephew, Memphis, TN). Sixteen cases that were identified were further analyzed and underwent screening for infection including determination of sedimentation rate and C-reactive protein (CRP) levels, as well as joint aspiration and culture. Figure 1 shows a typical case of a patient in whom such a local bone resorption was found.

**Figure 2:**
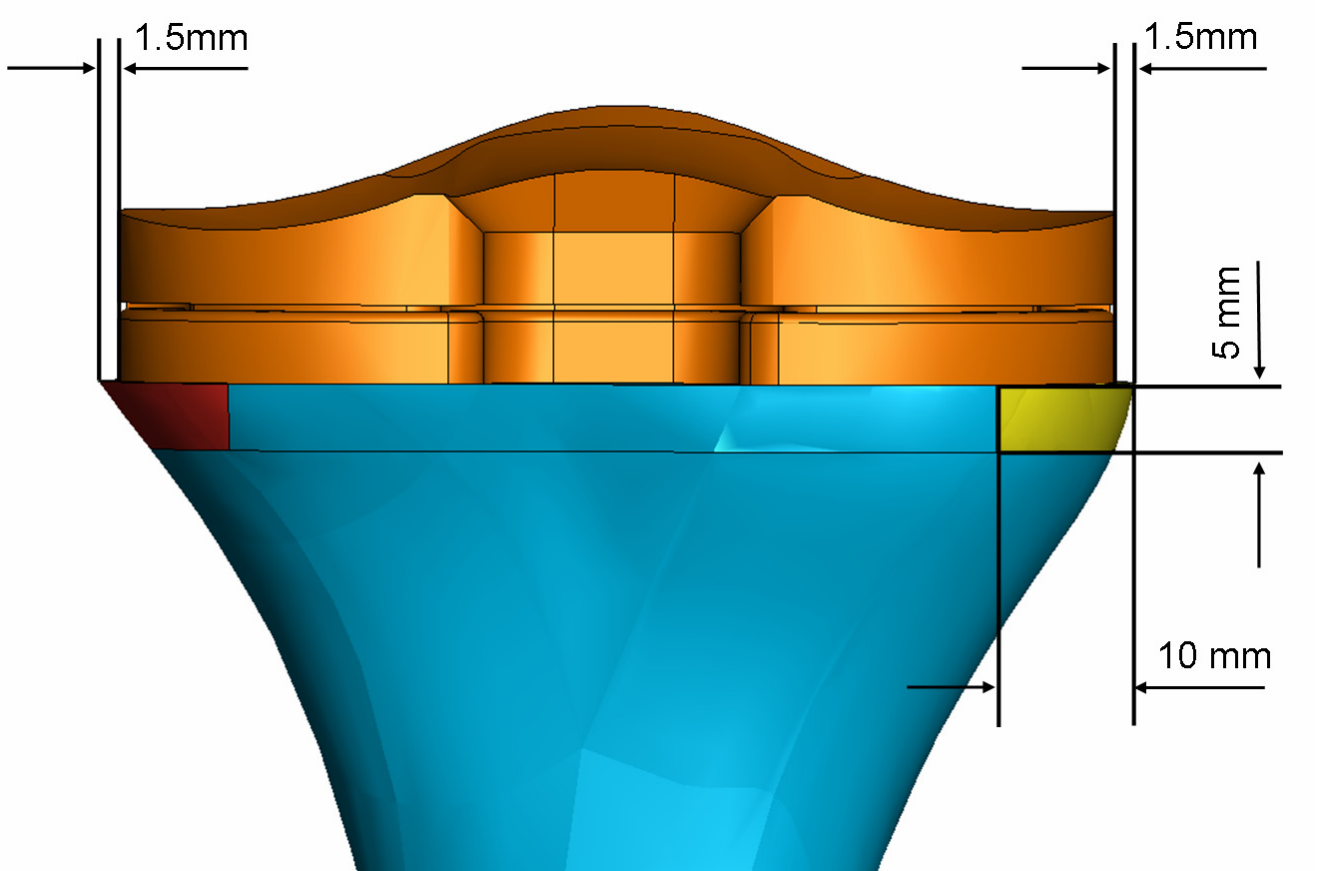
**Central position of the prosthesis in the tibia model; the baseplate is equidistant from the medial and lateral edges of the bone**. The medial and lateral regions of interest are dimensioned and colored. The picture shows only the proximal bone for clarity.

**Figure 3:**
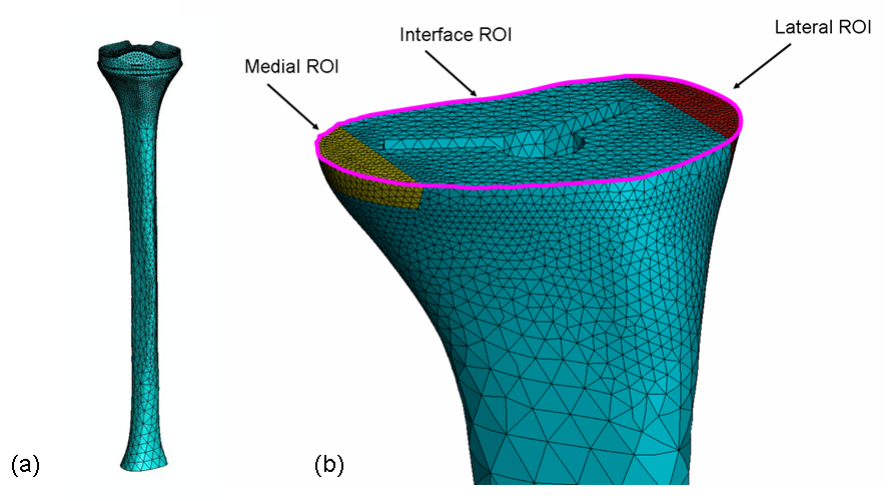
**Mesh of the three-dimensional Finite Element model of the prosthetized tibia**. a) Complete FE model mesh; b) Proximal view demonstrating the higher density of the mesh at the three ROI's, which are shown in different colors.

Based on the identified cases, a three-dimensional finite element model of the tibia with the prosthesis in situ was developed (Abaqus/Standard version 6.6-1, Abaqus, Inc., Providence, RI) from the "Standard Tibia" model [1,5,22-26]. This model was adapted for the implantation of a standard Genesis II asymmetric tibial Ti baseplate and its UHMWPE insert. A size 5 baseplate was chosen for this study since it is a common size used in clinical practice. The tibial bone model was scaled such that the resected proximal bone surface would be 3.0 mm larger in the mediolateral (ML) direction than the baseplate (Figure 2). Three regions of interest (ROI) in the tibia model were defined: the bone-baseplate interfacial ROI, the medial periprosthetic ROI, and the lateral periprosthetic ROI (Figures 2, 3). Based on the 16 patients' radiographs, the dimensions of each periprosthetic ROI were chosen to be 10 mm wide mediolaterally and 5 mm high. Each ROI had an anteroposterior length that spanned the bone (Figures 2, 3).

The baseplate was implanted on the tibia, following the surgical technique at an 11 mm tibial resection level perpendicular to the intramedullary canal [27]. The baseplate was placed initially in a central mediolateral position, equidistant from the medial and lateral edges of the bone (Figure 2). To simulate the presence or not of the cement between the tibial insert and the bone, two different models were defined. In the cementless model an interface gap of 1 mm was left between the cancellous bone and the stem/fin construct. When the implant was shifted to the medial or to the lateral side, this interface gap around the stem/fin construct was repositioned accordingly. Figures 2, 3 show example tibial constructs. To consider the effect of the cement, a layer of bone cement of 4 mm was considered between the bone and the baseplate and no gap was considered between the two structures [28,29].

Although cortical and cancellous bone show viscoelastic properties, the assumption of linear elasticity is adequate for most studies [1,5,24,30-34]. Accordingly, in this study, bone was assumed to be linearly elastic and isotropic. The material properties and behavior of the cortical bone, cancellous bone and titanium alloy are shown in Table 1[1,34,35]. The UHMWPE was assumed to be a non-linear elastic-plastic material according to the literature (E = 685 MPa, υ = 0.4, [36-41]). Also the cement layer was assumed to have linear elastic material properties (E = 3.0 GPa, υ = 0.3, [42-46]).

**Table 1:** Material properties and material behaviour used in this study [1,30].

**Material**	**Young's Modulus E (GPa)**	**Poisson's ratio**	**Material behavior**
Cortical Bone	16.6	0.3	Homogeneous, linearly elastic, isotropic

Cancellous Bone	2.4	0.3	Homogeneous, linearly elastic, isotropic

Titanium Alloy (Ti6Al4V)	117	0.3	Homogeneous, linearly elastic, isotropic

Based on literature a coefficients of friction of 0.15 was chosen for the insert-baseplate interface, a coefficient of friction of 0.2 was chosen for the bone-baseplate interface and a coefficients of friction of 0.3 was chosen for the cement-baseplate and cement-bone interface [42-46].

**Figure 4:**
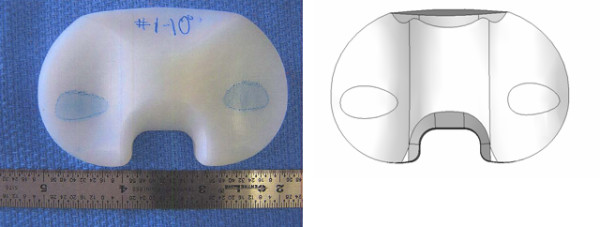
**Contact area's as found experimentally (on the left side), and contact areas used in the numerical model (on the right)**.

A static load of 800 N, corresponding to average body weight, was applied on two contact areas placed on the lateral and the medial condyles. The load was shared between the two areas in eleven load distributions, ranging from 0 to 100% (100 to 0%) on the lateral (medial) condyle with steps of 10%. The load on each area was distributed homogenously and perpendicularly to the baseplate for each load sharing configuration. To identify the magnitude of the loading contact areas on the polyethylene, a static experimental test was performed on a same size Genesis II femoral component against a size 5–6 tibial insert using a dye method and a loading frame. Based on this study, ellipsoidal contact areas on the medial and lateral condyles were created on the insert with magnitudes of 121 mm^2 ^and 132 mm^2^, respectively in the same position as seen in the experimental study. Figure 4 shows the result of the experiment and also the location and the magnitude of the contact area used for the numerical model.

For each load sharing configuration, seven baseplate positions were simulated:

1. the central ML position (Figure 2);

2. 0.5 mm medial displacement from the central position;

3. 1 mm medial displacement from the central position;

4. 1.5 mm medial displacement from the central position (the medial edge of the prosthesis component is in contact with the medial edge of the bone);

5. 0.5 mm lateral displacement from the central position;

6. 1 mm lateral displacement from the central position;

7. 1.5 mm lateral displacement from the central position (the lateral edge of the prosthesis component is in contact with the lateral edge of the bone).

The tibial bone model was trimmed distally and the cut section was considered fixed in all the simulations (Figure 3). The entire model was meshed with approximately 55,000 modified 8-node tetrahedral elements; the mesh density was increased for the three ROIs (Figure 3). Convergence of the FEA was checked using several mesh densities ranging from 5,000 up to 90,000 elements for two different configurations (central ML and 1 mm lateral position of the baseplate both with 50% load on each condyle).

One hundred-fifty-four simulations were run in total (11 load sharing conditions – 7 positions – cementless and cemented techniques). For each simulation, the average VonMises stress in each ROI was evaluated and plotted versus lateral load share and implant position.

## Result

### Clinical and radiographic observation

Of the 500 patients analyzed, only 16 cases (3.2%) showed local bone resorption of the proximal tibia. Resorption was seen on the medial side only. In each of these, the bone loss became apparent on radiographs at 3 months follow-up (Figure 1) and did not increase after one year. None of these cases were clinically symptomatic, and all 16 patients had "good" to "excellent" knee pain and function scores. Infection screening including joint aspiration was negative for all. Postoperative full leg radiographs demonstrated an overall mechanical alignment of neutral ± 3 degrees for all cases. Analysis of the pre-, per- and postoperative parameters of these 16 cases did not show any significant difference in either in parameters related to the preoperative status or diagnosis, the operative technique, implant specifications, or postoperative radiographic data when compared to the average of our database. No obvious difference between knee alignment and overall morphology of the knees in this 16 patients compare top the overall population. The only consistent finding in retrospect on these 16 cases was the absence of tibial cortical rim contact on the medial side, due to either an undersized tibial baseplate or a somewhat lateralized position of the baseplate.

### FEA Results

#### Convergence

**Figure 5:**
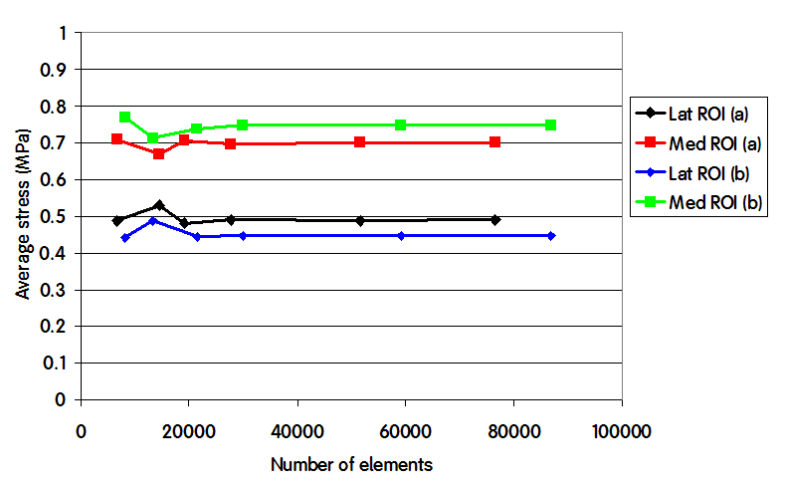
**Average VonMises stress in the Lateral ROI and in the Medial ROI for different number of elements in two configurations**. a) central ML baseplate position, 50% load on each condyle; b) 1 mm lateral displacement of the baseplate from the central position, 50% load on each condyle.

The results of the convergence test are shown in figure 5. The average Von Mises stress for the lateral and medial ROIs is constant for all meshes above 30,000 elements.

#### Interface ROI

**Figure 6:**
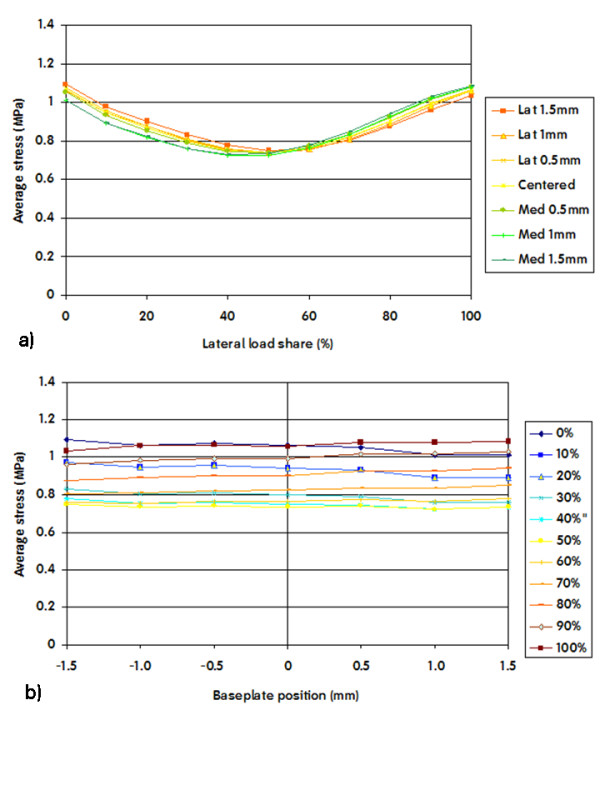
**Average VonMises stresses in the interfacial ROI plotted versus load share (a) and baseplate position (b)**. A 0% lateral share means that the entire load was applied on the medial area. A negative baseplate positioning means a shift of the component towards the lateral side.

The results of the finite element analysis demonstrated that stress in the interfacial ROI was lowest when load sharing was equal between medial and lateral. (Figure 6a) The average stress in the interfacial ROI was not influenced by mediolateral baseplate position. (Figure 6b)

#### Lateral ROI

**Figure 7:**
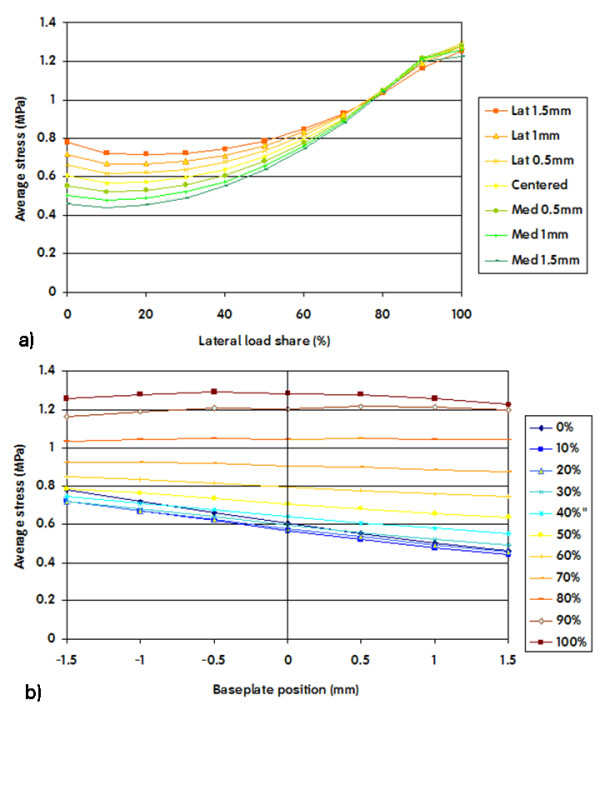
**Average VonMises stresses in the lateral ROI plotted versus load share (a) and positioning (b)**. A 0% lateral share means that the entire load was applied on the medial area. A negative baseplate positioning means a shift of the component towards the lateral side.

Stress in the lateral ROI increased significantly when the load was predominantly lateral, and decreased when the load was progressively shared with the medial side. (Figure 7a) This finding was dependent on implant position, with a greater decrease in stress on the lateral ROI when the tibial component was shifted medial.

Shifting the baseplate medial away from the lateral cortex caused reduced stress on the lateral ROI, especially in conditions of important load sharing towards the medial side (Figure 7b).

#### Medial ROI

**Figure 8:**
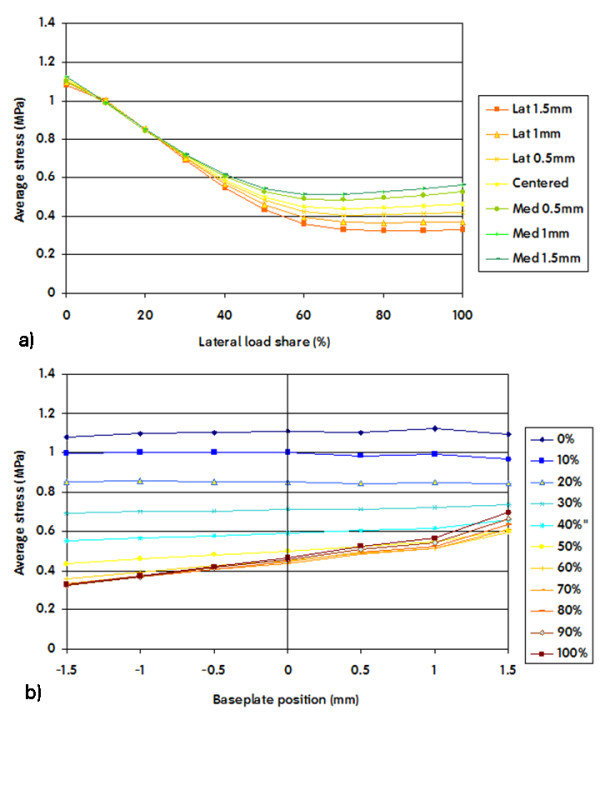
**Average VonMises stresses in the medial ROI plotted versus load share (a) and positioning (b)**. A 0% lateral share means that the entire load was applied on the medial area. A negative baseplate positioning means a shift of the component towards the lateral side.

Stress in the medial ROI increased significantly when the load was predominantly medial, and decreased when the load was progressively shared with the lateral side. (Figure 8a) This finding was again dependent on implant position, with a greater decrease in stress on the medial ROI when the tibial component was shifted lateral.

Shifting the baseplate lateral away from the medial cortex caused reduced stress on the medial ROI, especially in conditions of important load sharing towards the lateral side (Figure 8b).

#### Effect of cement layer

**Figure 9:**
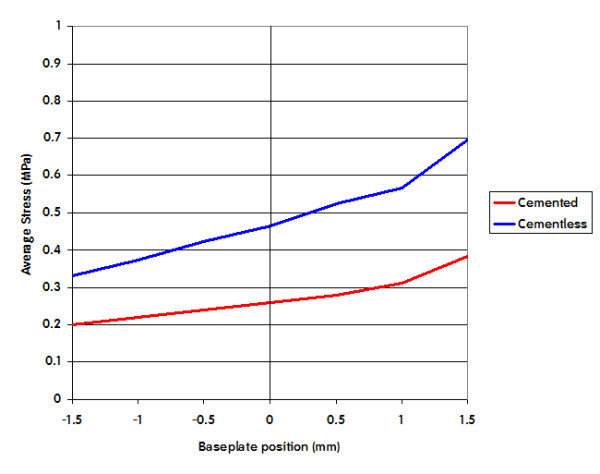
**Effect on the Average VonMises stress due to cement technique**. In this picture is shown the Average Stress in the medial ROI for 100% of load sharing for cemented and cementless technique.

The use of a cement layer between the tibial component and the bone induced a general reduction of the stress in all the ROIs. The overall trends described above remain valid. Figure 9 shows an example of the average VonMises stress in the medial ROI for cemented and cementless techniques under the same load condition.

#### ROI comparison

If we compare the variation of the average VonMises stress in the lateral ROI as a function of load sharing (Figure 7a) we see that the maximum variation is about 0.76 MPa (max = 1.22 MPa, min = 0.46 MPa). Similarly if we compare the variation of the average VonMises stress in the medial ROI as a function of load sharing (Figure 7a) we see that the maximum variation is about 0.75 MPa (max = 1.08 MPa, min = 0.33 MPa).

If we compare the variation of the average VonMises stress in the lateral ROI as a function of position (Figure 7b) we see that the maximum variation is about 0.26 MPa (max = 0.71 MPa, min = 0.45 MPa). In contrast, if we compare the variation of the average VonMises stress in the medial ROI as a function of position (Figure 8b) we see that the maximum variation is about 0.37 MPa (max = 0.70 MPa, min = 0.33 MPa). Although this variation doesn't seem very high in absolute values, the relative variation in the medial and the lateral ROI is considerably different.

## Discussion

**Figure 10:**
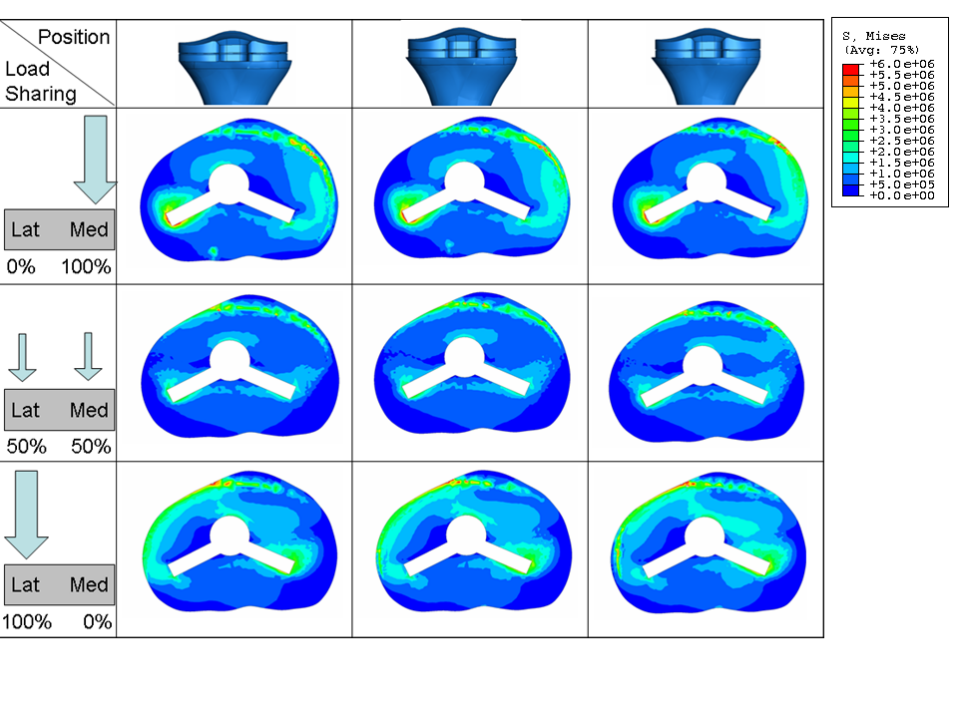
**VonMises stress distribution on the interface area for different positions (a) and load sharing (b) conditions**.

In this paper we present radiographic evidence of short term local bone resorption. This phenomenon is clinically asymptomatic and occurs in about 3% of the patients. It cannot be attributed to infection or wear and therefore we investigated possible mechanical reasons such as medio-lateral load distribution and baseplate position. A finite element analysis of a tibia implanted with a tibial knee arthroplasty baseplate was performed to investigate the stress distribution in the proximal tibial metaphysis. Three regions of interest were evaluated: the total interface between bone and tibial component (interfacial ROI), the medial periprosthetic region, as well as the lateral periprosthetic region of the proximal tibial metaphysis. Cemented and cementless techniques were considered. Our results demonstrate that in the interfacial ROI the average stress was not sensitive to implant position, but was moderately sensitive to load sharing (Figure 6a). Higher average stress values were obtained when the load was exclusively applied on one condyle, and lower values were observed when the load was shared more evenly amongst both compartments. This finding is logical, since application of the load on only one condyle generates a moment that disappears when the load is evenly distributed between both condyles. Figure 10 clearly demonstrates this phenomenon: when load is applied exclusively medially or laterally (Figure 10 left row and right row) the stress concentrates near respectively the lateral and the medial edge of the stem/fin construct, whereas an equally distributed load amongst both condyles (Figure 10 middle row) will generate no moment and therefore also lower the stress on the edges.

**Figure 11:**
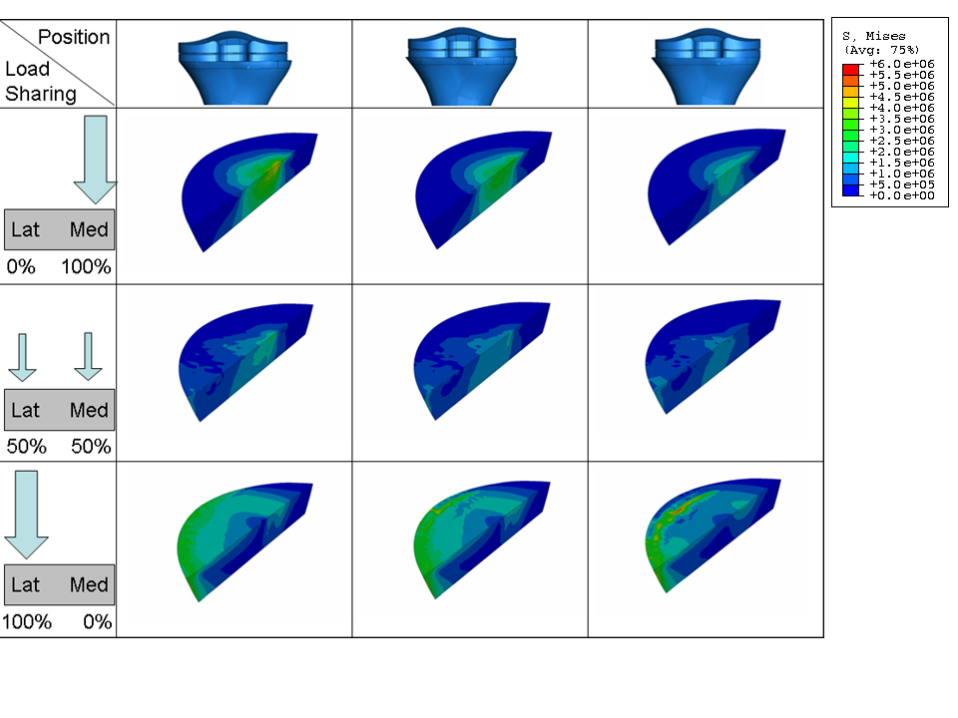
**VonMises stress distribution on the Lateral ROI for different position (a) and load sharing (b) conditions**.

The average stress in the lateral ROI as a function of load sharing (Figure 7a and Figure 11) is almost constant as long as the lateral condyle carries less than 50% of the load. When the lateral condyle carries more than 50% of the load, the stress in the lateral ROI increases linearly. Stress in the lateral ROI presented a linear relationship with implant position (Figure 7b and Figure 11) for all of the considered load distributions. However, the slope of this linear relationship is not constant. It is steeper as soon as less than 50% of the load is carried by the lateral condyle.

**Figure 12:**
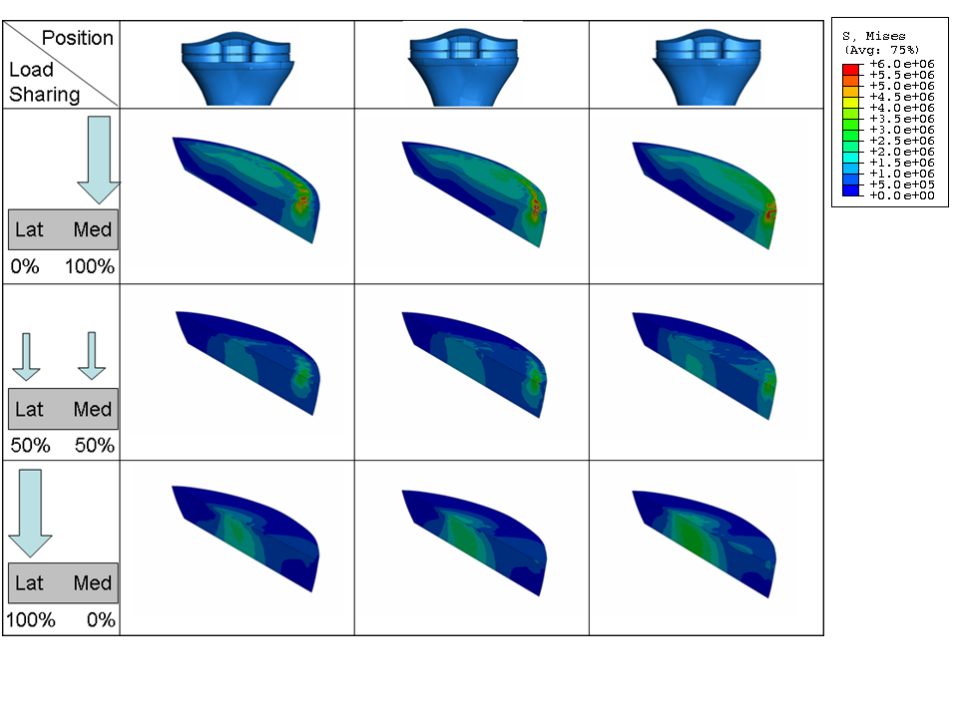
**VonMises stress distribution on the Medial ROI for different position (a) and load sharing (b) conditions**.

The average stress in the medial ROI as a function of load sharing (Figure 8a and Figure 12) behaves almost identical as in the lateral ROI. It is almost constant as long as the medial condyle carries less than 50% of the load, when the condyle carries more the stress increases linearly.

In contrast, the average stress in the medial ROI as a function of implant position (Figure 8 and Figure 12), presents different behaviour compared to the stress in the lateral ROI.

The average stress in the medial ROI changes non linearly when the component is placed near the medial edge of the cortex and the medial condyle carries less than 50% of the load. This change becomes linear as soon as the component is shifted more than 1 mm away from maximal medial cover (Figure 8b).

This change is also much less dramatic when load is greater than 50% on the medial compartment, such as one could expect in case of a varus alignment. This again is a logical finding. Medial compartment load is high in the varus situation, and shifting away the tibial component lateral from the medial cortex will not lead to an important reduction of stress on the medial side. In the valgus situation however, load on the medial compartment is already reduced, and shifting the component away from medial cortex will further reduce the medial tibial stress in an important way.

Simulation of cemented fixation of the baseplate shows similar trends as in a cementless fixation.

FEA results indicate that the tibial positioning is more important than the load distribution on the VonMises stress in the medial tibial ROI. This is in agreement with the full leg radiographs that show proper alignment, and thus normal load sharing, in the 16 cases where local bone resorption occurs.

Based upon our findings the average stress in the medial ROI seems therefore more sensitive than the lateral ROI to implant position and to a certain extent also to load sharing.

If we compare the decrease of the average stress in the medial and lateral ROI for 1 mm shift of the component away from the respective cortex in the range of the physiological load sharing condition (60% of the load on the medial condyle), we obtain a decrease on the medial ROI which is more than 50% greater than the decrease in the lateral ROI (stress in medial ROI changes 0.19 MPa/mm, stress in lateral ROI changes 0.12 MPa/mm, Figure 7b, Figure 8b). Although the variation of the average VonMises stress in the lateral and the medial ROI doesn't seem very high in absolute values (0.26 MPa and 0.37 MPa respectively), the relative variation in the lateral and the medial ROI is very different (45% and 72% respectively).

We believe that our finite element data shows that important stress relief can easily occur on the medial side when the tibial implant is not positioned on the medial cortex, and that this could lead to unloading the medial cortex. Our finite element data also shows that the lateral cortex is much less subject to stress deprivation in case the tibial component is not or less in contact with the lateral cortex. The reason for this may be the fact that the lateral tibial cortex is much less pronounced and solid compared to the medial cortex, and therefore may much less contribute to stress transfer. Although our FEA is not sufficient to explain completely the local asymptomatic bone resorption phenomena that we found in our clinical observations; these results may contribute to an explanation and certainly warrants further investigations. Even if the leg alignment in the 16 patients was normal, or at least not abnormal, only more detailed in vivo analysis can evaluate the exact load sharing condition in these knees to allow the confirmation of our finite element analysis. Also the influence of other parameters (i.e. thickness of tibial baseplate, symmetric geometry of the tibial tray) and the effect of other positioning factors (slope, varus-valgus and rotation) will be further examined.

## Competing interests

Bernardo Innocenti, Luc Labey and Pius Wong are all employees of the Smith & Nephew Company. They are all working at the European Centre for Knee Research (Smith&Nephew) in Leuven, Belgium.

Jan Victor and Johan Bellemans are both consultant for the Smith & Nephew Company and supervised studies in the European Centre for Knee Research, Leuven, Belgiun.

## Authors' contributions

All the authors participated in the design of the study. ET, JB carried out the radiographic analysis. BI, PW carried out the Finite element analysis. All the authors helped to draft the manuscript. All authors read and approved the final manuscript.
